# Pigmented villonodular synovitis of the hip joint: Three cases demonstrating characteristic MRI features

**DOI:** 10.1016/j.radcr.2020.05.067

**Published:** 2020-06-24

**Authors:** Charles J. Sullivan, Stephen J. Eustace, Eoin C. Kavanagh

**Affiliations:** a Department of Radiology, National Orthopaedic Hospital Cappagh, Finglas, Dublin 11, Ireland; b Department of Radiology, Mater Misericordiae University Hospital, Eccles Street, Dublin 7, Ireland

**Keywords:** Pigmented villonodular synovitis, Synovium, Hip, MRI, Radiology, Case reports

## Abstract

Pigmented villonodular synovitis is a rare benign proliferative disease of synovial membranes, causing villonodular synovial hyperplasia and hemosiderin deposition. Its intra-articular forms most commonly affect the knee and less commonly the hip. PVNS of the hip is most common in the second to fifth decades and presentation is typically with pain and occasionally joint dysfunction. We review the existing literature and demonstrate characteristic magnetic resonance imaging features of pigmented villonodular synovitis in the hip joint using three biopsy-proven cases, with the aims of increasing awareness and aiding diagnosis of this rare but potentially debilitating and progressive condition. Recognition of its clinical presentation, appropriate use of magnetic resonance imaging and identification of imaging characteristics are essential to guiding biopsy interpretation and treatment.

## Case report

Review of pigmented villonodular synovitis (PVNS) cases at our institution revealed three patients with PVNS of the hip joint, each of whom had been referred for specialist opinion from outside institutions following unremarkable radiographs. Images from each case were selected for illustration of magnetic resonance imaging (MRI) features.

**Fig. 1:**
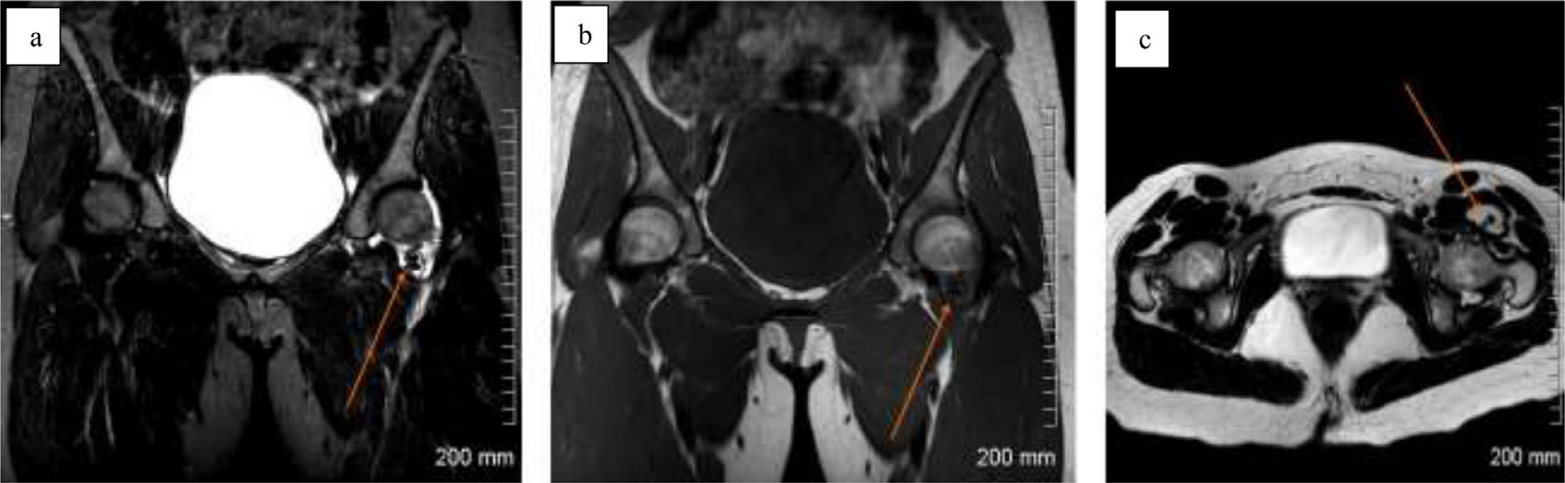
Case 1. Pelvic MRI sequences in a 15-year-old female. MRI demonstrates a moderate left hip effusion of intermediate T1 signal, implying hemosiderin content, with hypointense filling defects in the anterior recess of the joint. (a) Coronal short tau inversion recovery (STiR) sequence of the pelvis with left hip effusion (arrow), (b) coronal T1 sequence with left hip effusion (arrow), (c) axial T2 sequence of the pelvis with left hip effusion (arrow).

Case 1: A 15-year-old female with no relevant past medical history presented initially with a 6-day history of left hip pain and limping. Physical examination demonstrated reduced straight leg raise on the left side and reduced active and passive range of motion at the left hip with a block to internal and external rotation. The remainder of the physical examination was normal and the patient was afebrile. Laboratory investigations were unremarkable apart from a C-reactive protein of 13. Pelvic radiograph showed no abnormality. MRI demonstrated a moderate left hip effusion of intermediate T1 signal, implying hemosiderin content, with hypointense filling defects in the anterior recess of the joint (Fig. 1). Ultrasound-guided synovial core biopsy was performed and diagnosis of PVNS was confirmed at histopathologic examination.

**Fig. 2:**
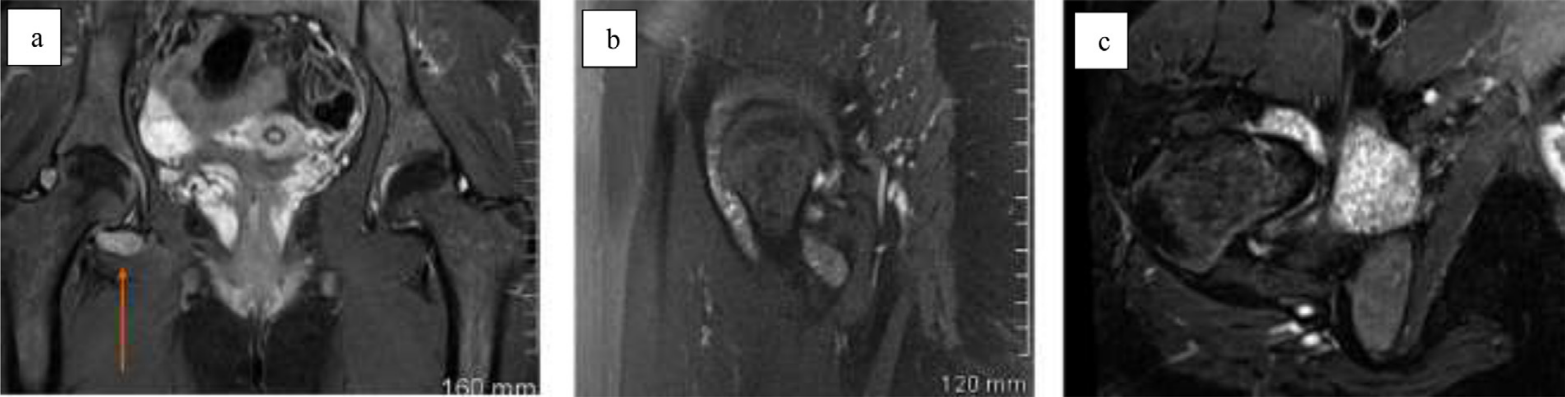
Case 2. Pelvic MRI sequences in a 26-year-old female. MRI demonstrates a moderate right hip joint effusion with multiple hypointense intra-articular bodies. (a) Coronal STiR sequence, (b) sagittal PD fat-suppressed sequence of the right hip, (c) axial T2 fat-suppressed sequence of the right hip.

Case 2: A 26-year-old female with no relevant past medical history presented with right hip pain, limping and subjective decrease in range of right hip motion, all of which had progressed over the previous 18 months. Physical examination demonstrated reduced active and passive range of motion at the right hip with associated pain. The remainder of the physical examination was normal and the patient was afebrile. Laboratory investigations were normal. Pelvic radiograph showed no abnormality. MRI demonstrated a moderate right hip effusion with multiple hypointense intra-articular bodies and no evidence of erosion or donor sites (Fig. 2). Ultrasound-guided synovial core biopsy was performed and diagnosis of PVNS was confirmed at histopathologic examination.

**Fig. 3:**
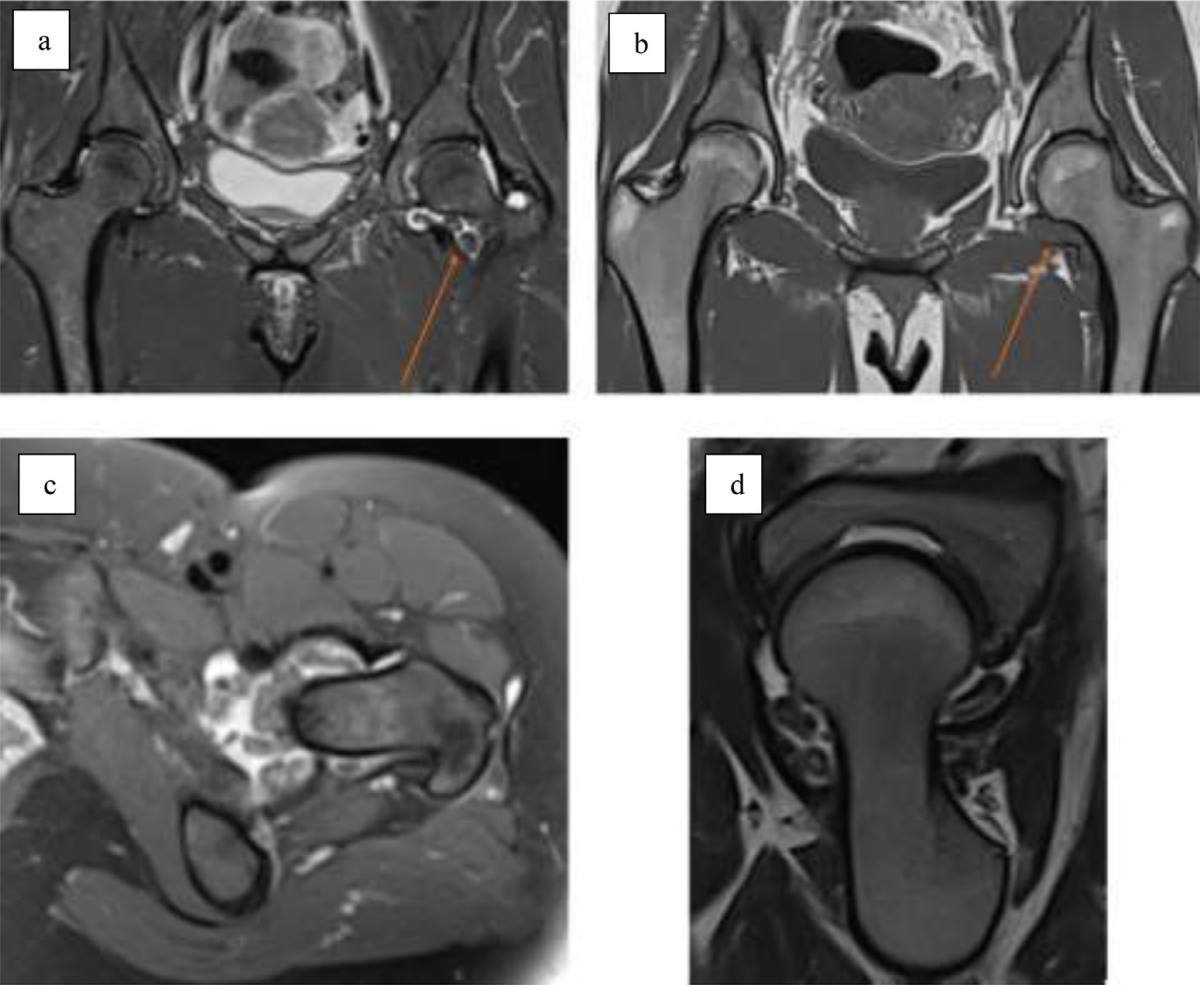
Case 3. 38-year-old female. MRI demonstrates a moderate left hip joint effusion with multiple hypointense intra-articular bodies. (a) Coronal STiR sequence of the pelvis with left hip effusion (arrow), (b) coronal T1 sequence of the pelvis with left hip effusion (arrow), (c) axial PD fat-suppressed sequence of the left hip, (d) sagittal T2 sequence of the left hip.

Case 3: A 38-year-old female with no relevant past medical history presented with a six-month history of progressive left hip pain and limping. Physical examination demonstrated reduced active and passive range of motion at the right hip with associated pain. The remainder of the physical examination was normal and the patient was afebrile. Laboratory investigations were normal. Pelvic radiograph showed no abnormality. MRI demonstrated a moderate left hip effusion with multiple hypointense intra-articular bodies (Fig. 3). Ultrasound-guided synovial core biopsy was performed and diagnosis of PVNS was confirmed at histopathologic examination.

## Discussion

PVNS is a benign proliferative disease of synovial membranes causing villous, nodular or villonodular synovial hyperplasia and hemosiderin deposition. The current descriptions of the disease were published by Jaffe et al. in 1941 who distinguished the types of PVNS by site of involvement, namely pigmented villonodular synovitis, bursitis and tenosynovitis [1]. The World Health Organization has since updated the nomenclature to describe the localized extra-articular bursitis and tenosynovitis forms of PVNS as giant cell tumour of the tendon sheath, and the diffuse intra-articular form of PVNS as diffuse-type giant cell tumour [1,2]. Intra-articular PVNS is a rare condition with average annual incidence estimated at 1.8 per million people [3]. Diffuse intra-articular PVNS is most common in the third to fifth decades, with mean age in the late 30s, and occurs with equal frequency in both sexes [4]. It is almost invariably monoarticular [5]. Diffuse intra-articular PVNS affects the knee in 66%-80% of cases, the hip in 4%-16% of cases, and less frequently other large joints such as the ankle, shoulder and elbow [4].

Pain and swelling are typical symptoms of intra-articular PVNS. Joint dysfunction occurs less frequently and a soft tissue mass is unusual [4]. Duration of symptoms has been reported to vary widely, from one to 120 months with a mean of 15 months for diffuse intra-articular disease, and with chronic intermittent symptoms in most cases [3].

Radiographs in the context of diffuse intra-articular PVNS may appear normal in up to 21% of patients, as in the above cases, but more often demonstrate joint effusion or extrinsic bone erosion with preservation of joint space and normal bone mineralization [6,7]. Erosive changes of PVNS are seen in 93% of cases of hip PVNS [8] due to smaller capacity of the hip joint relative to the knee and absence of adjacent bursae that would allow decompression and extension of synovial tissue out of the joint. Less frequent radiographic features of diffuse intra-articular PVNS include osteopenia, joint space narrowing, osteochondral bodies and degenerative disease.

Although not performed in the cases shown, ultrasound and computed tomography (CT) can suggest the presence of diffuse intra-articular PVNS but are less sensitive and less specific than MRI. Sonographic features such as heterogenous intra-articular masses and thickened hypoechoic synovium [5] may be amenable to ultrasound-guided core biopsy. CT may show extrinsic bone erosion and thickened synovium, which can be of increased attenuation relative to muscle secondary to hemosiderin deposition [9].

MRI of diffuse intra-articular PVNS demonstrates diffuse heterogenous synovial thickening that is often nodular, villous or villonodular in appearance, with a joint effusion. The thickened synovium demonstrates intermediate to low signal on T1-weighted imaging and low signal on T2-weighted imaging, secondary to T2 shortening caused by hemosiderin. Signal hypointensity is more apparent on gradient echo imaging, if performed, due to “blooming” or enlargement of the hypointense areas caused by magnetic susceptibility artefact, which indicates the presence of hemosiderin and is highly suggestive of PVNS. Differential diagnosis of this appearance could include hemophilic arthropathy, excluded by appropriate clinical history, and synovial hemangioma, which would demonstrate tortuous vascular channels [5,8]. PVNS commonly enhances following intravenous administration of gadolinium [5], which was not performed in the cases shown. Additional MRI features include bone erosion, subchondral cysts, septations, bone or soft tissue edema, and defects in articular cartilage [10].

Biopsy of PVNS is essential for diagnosis. The histologic appearance of diffuse intra-articular PVNS can resemble a more aggressive neoplastic entity such as synovial sarcoma, rhabdomyosarcoma or epithelioid sarcoma [2,^8^,11]. Correlation of the histologic features with the clinical history and imaging findings of diffuse synovial hyperplasia is therefore important in correctly diagnosing a benign process. An ultrasound-guided biopsy approach is preferable in our experience due to its minimally invasive nature and ability to visualize synovium. Treatment of PVNS aims to prevent progressive loss of function and joint destruction in intra-articular disease. Surgical resection is the treatment of choice for PVNS, with complete synovectomy necessary in the context of diffuse intra-articular disease. Recurrence is more common in diffuse-intra-articular PVNS and may require adjuvant radiation therapy, either by external beam radiation or intra-articular injection of a radioactive isotope. Recurrence rates of diffuse intra-articular PVNS following initial treatment vary from 8% to 56% [5,^12^,13]. Treatment strategies for recurrent diffuse intra-articular PVNS include combination therapies, joint replacement or rarely amputation in cases of multiple recurrences [12,13].

## Conclusion

MRI is the most appropriate imaging modality for detection and assessment of disease extent in PVNS. Recognition of its clinical presentation, appropriate and timely use of MRI and identification of MRI characteristics are essential in order to guide biopsy interpretation and successfully treat this rare but potentially debilitating and progressive condition.

## Declarations of interest

None.
